# Addressing the dual-use of antifungals and fungal antimicrobial resistance (fAMR) through a One Health approach

**DOI:** 10.1038/s44259-026-00220-9

**Published:** 2026-05-29

**Authors:** S. Weeks, A. Brown, K. Doughty, D. Armstrong-James, N. Bennett, M. Birch, A. M. Borman, M. Bromley, R. Bryson, M. C. Fisher, D. Freeman, M. Grimmer, J. H. Rex, H. West, P. L. White, M. J. Bottery

**Affiliations:** 1 School of Medical Sciences, University of Manchester, Manchester, UK; 2 Parliamentary Internship Programme, British Society for Antimicrobial Chemotherapy, Birmingham, UK; 3 School of Geography and the Environment, University of Oxford, Oxford, UK; 4 Crop Protection Consultant, Monheim, Germany; 5 Department of Infectious Diseases, Imperial College, London, UK; 6 Royal Brompton and Harefield Hospitals at Guy’s and St. Thomas, London, UK; 7 House of Lords, Parliament of the United Kingdom, London, UK; 8 F2G Ltd, Cheshire, UK; 9 UKHSA National Mycology Reference Laboratory, Bristol, UK; 10 Medical Research Council Centre for Medical Mycology, University of Exeter, Exeter, UK; 11 Manchester Fungal Infection Group, Division of Evolution, Infection and Genomics, University of Manchester, Manchester, UK; 12 RSK ADAS Ltd., Cambridge, UK; 13 Department of Infectious Disease Epidemiology, Imperial College, School of Public Health, London, UK; 14 Thames Water, Reading, UK; 15 Faculty of Science , University of Nottingham, Sutton Bonington Campus, Leicestershire, UK; 16 Mycology Reference Laboratory, Public Health Wales, Cardiff, UK; 17 Medical Microbiology, Division of Infection and Immunity, Cardiff University, Cardiff, UK

**Keywords:** Diseases, Health care, Microbiology

## Abstract

Fungal antimicrobial resistance (fAMR) is accelerating, driven in part by the dual-use of antifungal modes of action in agriculture and medicine, threatening therapy. Addressing this challenge requires a unified One Health response that balances agricultural productivity, economic stability, and human and animal health. By focusing on the United Kingdom’s policy approach, we argue that current efforts are constrained by fragmented governance, surveillance and regulation. To resolve this, we propose three policy recommendations: 1. a cross-government fAMR body, 2. mandatory environmental and clinical surveillance, and 3. for fungicide approvals to look beyond crop pathogens and integrate risk assessments for potential hotspots of resistance selection in human fungal pathogens. These measures will safeguard current and future antifungals while providing much-needed regulatory clarity and will be translatable to other national and regional contexts.

## The impact of the dual use of antifungals

Globally, fungi cause more than one billion superficial infections each year, while life-threatening invasive infections affect about 6.5 million people and result in approximately 2.5 million attributable deaths^1,2^. Infections due to *Aspergillus*, *Candida*, *Cryptococcus* and *Pneumocystis* species account for >90% of reported fungal-related deaths, while significant morbidity arises from chronic pulmonary aspergillosis, severe asthma with fungal sensitisation, allergic bronchopulmonary aspergillosis and superficial candidiasis^1,3^.

The burden of fungal antimicrobial resistance (fAMR) is increasing^4^ and resistant infections are consistently associated with worse outcomes^5^. For invasive aspergillosis, resistance to voriconazole is associated with poorer survival rates, reaching a 90-day mortality of up to 100%^6,7^. However, limited and often non-standardised surveillance means the population-level burden associated with fAMR remains uncertain^8,9^.

Systemic treatment for serious fungal disease relies on only three major antifungal classes: azoles, echinocandins, and polyenes. A fourth class, a pyrimidine analogue, is essential for cryptococcal and resistant *Candida spp*. Options for oral administration are limited, and many drugs are associated with significant adverse side effects, complex drug-drug interactions and poor availability in many low-resource settings^2,9,10^. Rising levels of resistance further undermine these often life-saving treatment options^3,9^. Azoles are the primary treatment option for many severe fungal infections^11^. As such, widespread environmental azole use is considered a key One Health driver of clinical risk. The emergence of azole-resistant human fungal pathogens has been linked to the extensive use of azole fungicides as plant protection products (PPPs) and biocides, which share the same mechanism of action as clinical azoles^9^.

Fungicides are widely used as PPPs in UK crop production, safeguarding the productivity of arable agriculture—which encompasses 37% of the total utilised agricultural area of the UK^12^—from plant diseases such as wheat yellow rust (*Puccinia striiformis)* and Septoria leaf blotch (*Zymoseptoria tritici*). According to the UK Pesticide Use Survey for the 2023–2024 season, fungicides were applied to 94% of UK arable crops, indicating their continuing, important role in maintaining yield and quality^13^. Plant diseases impose large and persistent production losses across the globe, with one estimate suggesting that treatment of currently uncontrolled, persistent disease in five major crops would have otherwise fed 8.5% of the global population in 2011^14^. Fungal mutations conferring resistance to fungicides threaten their efficacy and can thus increase yield losses: Resistance management is therefore essential for protecting yield and food security^15^. Such management of resistance to existing PPPs further depends on the ongoing availability of diverse modes of action, including innovative and novel fungicides.

Azole fungicides, a major subgroup of demethylation inhibitors (DMIs), are a core class in crop protection. They are applied as seed treatments, foliar sprays and post-harvest treatments to control damaging fungal diseases, and they play a significant role in disease management in all major crops^16^. Between 2010 and 2021, 99.2% of non-medical azoles sold in the EU were used as PPPs, while human consumption of medical azoles for systemic use was at least 1000 times lower, making crop protection the dominant azole consumer^11^.

The emergence of resistance to azole fungicides in target fungal pathogens of crops presents an ongoing challenge for disease management. For example, Septoria leaf blotch is one of the most economically significant leaf diseases in Western Europe due to its capacity to reduce yield in the absence of effective control measures^17^. Control of *Z. tritici* relies on treatment with fungicides, as cultural practices provide limited disease suppression^18^. However, this intensive use of azoles has coincided with well-documented but differential sensitivity loss to a number of azole DMIs within European *Z. tritici* populations. In the UK, the Agriculture and Horticulture Development Board’s (AHDB) monitoring and reporting has shown reduced sensitivity of Septoria to azole DMI fungicides, with continued variations across sites^19^. As such, the introduction of novel fungicides to the market, such as the azole DMI mefentrifluconazole, has been an important addition to *Z. tritici* control^20^, marking the significance of azole DMIs to agriculture in the UK.

Despite the importance of DMI fungicides in crop protection regimes, particularly where diseases have developed extensive resistance to alternative modes of action^16^, their widespread use has unintended consequences beyond target pathogen control. Non-target environmental fungi can be exposed to azole residues in agricultural environments^11,21^. Evidence suggests that such exposure can select for resistance in environmental *Aspergillus* spp., particularly the human pathogen *A. fumigatus*, and that these resistance mechanisms selected outside the clinic can confer cross-resistance to medical triazoles^11,21–23^. EU agencies conclude that azole use outside human medicine is likely or very likely to contribute to the selection of azole-resistant *A. fumigatus* isolates^11^ and several reviews identify specific environmental reservoirs and hotspots that may facilitate selection and dispersal^11,21,23^. Here, we define environmental ‘hotspots’ as environments that promote the selection, amplification and spread of resistance. For this to occur, (1) environmental conditions must facilitate the growth of the fungus, allowing it to achieve high growth rates and large population sizes; (2) fungicides are present at concentrations sufficient to select for resistance; (3) there is an opportunity for the biotic or abiotic dissemination of the fungus to at-risk human populations, or the wider environment.

In the UK, rates of resistance in *A. fumigatus* appear to be gradually rising in both the environment and the clinic^24^. In 2014, no azole-resistant *A. fumigatus* was observed in environmental samples from urban Manchester, whereas 1.7% of isolates were resistant in rural arable fields with long-term azole use, including TR34/L98H, the canonical environmental resistance mutation in the cyp51A gene^25^. In South Wales, a 5-month survey in 2015 found a 6.0% prevalence of azole resistance among environmental *A. fumigatus* isolates, with most resistant isolates carrying TR34/L98H^26^. More recently, citizen-science studies detected triazole-resistant *A. fumigatus* in 5% of isolates from air samples^27^ and 14% of isolates in garden soils^28^, showing routine exposure beyond occupational settings. Population genomic analyses show that UK clinical resistant isolates carrying TR34/TR46 are genetically very closely related to those found in environmental isolates^29^, indicating that background exposures can lead to clinical infections. Evidence from various settings supports the role of specific environmental hotspots in resistance selection. In the Netherlands, environmental hotspots such as flower-bulb waste piles, green-waste heaps and wood chippings show azole residues coinciding with dense *A. fumigatus* growth and high frequencies of resistance, predominantly the *cyp51A* TR34/L98H and TR46/Y121F/T289A genomic variants^30^. In contrast, low levels of pan-azole resistant isolates were detected in arable soils from wheat crops treated with azoles, suggesting these are agricultural coldspots^31^. More recent Dutch research relating frequencies of these variants in air samples to local land use patterns shows variation among different crops and cropping scenarios, with flowerbubs, greenhouses and potato crop settings as potential sites of resistance selection^32^.

Environmental azole-resistant *A. fumigatus* is observed globally^23,33^. The ability of *A. fumigatus* to produce vast numbers of aerosolisable spores within hotspots such as plant waste piles, which are able to travel large distances, enables the dispersal of resistance. In addition, international dissemination has been linked to the global trade of flower bulbs and other plant materials^34,35^. Resistance can be selected locally within hotspots and then spread beyond national borders, highlighting the need for coordinated surveillance and risk assessment across countries. This framing clarifies why best practices for agricultural waste management, nuanced use of azole fungicides and cross-resistance assessment during fungicide approval are relevant interventions within a One Health approach^11^. Climate change is likely to increase the pressure upon both antifungals and fungicides due to anticipated elevated mutation rates in pathogens, changes in pathogen range and increased frequency of extreme events^2,36–38^. Therefore, responsible stewardship of all antifungal agents, irrespective of use, is of paramount importance to safeguarding the essential roles these tools play in both human medicine and food security. The azole–*Aspergillus fumigatus* system provides the strongest empirical evidence for environmental fungicide selection driving clinically relevant resistance. The extent to which similar environmental selection drives cross-resistance among other fungal pathogens and antifungal/fungicide classes remains limited. Nevertheless, the underlying governance challenges associated with dual-use of antifungals are likely to extend beyond this system, allowing for the identification and implementation of mitigation strategies preemptively, prior to the emergence of widespread resistance.

fAMR represents a major policy and research gap which requires urgent attention. Only recently have the WHO Fungal Priority Pathogens List (FPPL) and the WHO global AMR research agenda formalised fungal pathogens as central to the AMR crisis, highlighting gaps in surveillance, diagnostics and funding^3,8,10^. While the WHO FPPL provides an important global framework for prioritisation, the relative burden of fungal pathogens varies geographically, and priority rankings may not fully reflect region-specific disease patterns. Local epidemiology should therefore inform pathogen prioritisation and the design of surveillance and policy responses. However, many AMR strategies, including the Global Action Plan on AMR, still emphasise bacterial threats, with fungal risks and their environmental or agricultural drivers receiving less attention^2,9^. Recognising fAMR as a distinct One Health challenge is therefore critical to developing a targeted governance response^9^.

A 2019 One Health workshop in Amsterdam highlighted the need for coordination between healthcare professionals, pharmaceutical companies, agricultural scientists, growers, composting companies, regulators, food retailers and the crop-protection industry to address environmental azole use and *A. fumigatus* resistance^21^. This approach has been formalised in an European Food Safety Authority (EFSA)-led multi-agency assessment framed as a One Health call for action on the risks deriving from environmental use of azoles^11,39^.

A similar approach is needed in the UK. However, governance of antifungal dual-use would span pesticide regulation, agriculture, environmental management and clinical AMR, and there is no governing body mandated to coordinate these domains or assess how fungicide decisions affect resistance risks in human pathogens. We argue that progress on fAMR now depends on aligning governance structures with the One Health pathways through which resistance emerges, and we outline recommendations to make fAMR actionable within pesticide and AMR policy. This governance gap is most visible with respect to surveillance, in which clinical and environmental monitoring remain poorly integrated, limiting the UK’s ability to detect emerging fAMR risks, attribute environmental drivers and evaluate mitigation measures over time. While we focus on the UK as a case study, many of these governance gaps are systemic and globally prevalent; antifungal resistance surveillance remains limited worldwide, and to the best of our knowledge, no existing pesticide regulatory process currently assesses the risk of cross-resistance in human fungal pathogens.

## Surveillance gaps

UK fAMR surveillance remains fragmented across clinical and environmental domains, which is likely to limit attribution and timely detection of resistance. In the UK, clinical antifungal resistance surveillance is largely delivered through specialist mycology reference laboratories and centres, but practice and coverage remain heterogeneous and often rely on voluntary participation^5^. Environmental surveillance for azole-resistant *A. fumigatus* remains limited and ad hoc; EU agencies report the absence of monitoring and surveillance, calling for standardised prevalence studies at national and EU levels across the human, animal and environmental domains^11^. Key gaps include sparse monitoring of agricultural azole residues in potential transmission networks for the spread of drug-resistant strains, such as soil and water, alongside limited evidence on the hotspot conditions and waste-management practices that promote the selection of resistance^11,21^. These limitations will constrain regulators’ ability to attribute resistance selection to defined agricultural fungicide use cases and to assess whether changes to pesticide use or waste handling can reduce selection pressure over time. Addressing this will require systematic sampling that links residue measurements with *A. fumigatus* growth and resistance frequencies^11,21,30^.

This gap is recognised in international and UK agendas. The WHO global AMR research agenda prioritises improved surveillance and burden estimates for fungal pathogens^8^. The UK fAMR roadmap likewise calls for integrated One Health surveillance, including monitoring environmental reservoirs and resistance hotspots^9^. The UK Chief Medical Officer’s 2025 annual report similarly warns that agricultural use of novel fungicides may drive the spread of resistant fungi capable of causing serious and potentially hard-to-treat human disease, and calls for research and surveillance without specifying how this should be implemented^40^.

Across WHO priority human fungal pathogens, evidence on burden and resistance remains sparse and regionally uneven, reflecting variable access to diagnostics and susceptibility testing^8,10^. Consistent with this, a workshop-based Northern European perspective reports that nationwide surveillance data for *Candida* spp are accessible in several high-income countries, though not always systematic, whereas surveillance for azole-resistant *A. fumigatus* and other priority human pathogens is less common and constrained by variation in diagnostic access and standardisation across countries^3^. Taken together, we argue this supports the case for strengthening and standardising surveillance beyond the best-covered syndromes to enable earlier detection of emerging fAMR and to make One Health monitoring actionable. This need applies equally to the UK. Where robust clinical datasets exist, they are rarely integrated with environmental measurements of resistance. Environmental studies documenting the prevalence of azole-resistant *A. fumigatus* in rural arable fields, compost, green-waste, garden soils and bioaerosols, including TR34/TR46-positive hotspots, are typically disconnected from routine clinical surveillance and largely limited to rare specialist genomic analyses^21,25–27,29,30,41,42^. Such separation is inconsistent with One Health principles, which will limit policymakers’ ability to trace environmental–clinical resistance pathways and detect emerging threats early. This is despite integrated surveillance being identified as a priority in a recent fAMR roadmap 9 and by the UK Chief Medical Officer^40^.

The UK has played a leading role in shaping the global antibacterial AMR agenda and invests heavily in infrastructure that could, in principle, support integrated fungal surveillance, including national reference laboratories, genomic platforms and environmental monitoring schemes^9,40^. For example, the PATH-SAFE programme is developing a national system for pathogen sequencing and environmental monitoring across agriculture, food and the environment, and it has begun to explore antifungal resistance selection in soil and water and genomic tracking of *Candida auris*^9^. The UK fAMR roadmap also sets out practical steps such as piloting environmental and clinical surveillance around known hotspots, incorporating fungi into AMR dashboards and using genomics to link environmental and patient isolates^9^. Implementing these approaches would strengthen the empirical foundation needed to support regulatory reform and One Health governance for fAMR.

## Policy needs in the UK

Environmental selection of antifungal resistance exemplifies a One Health problem, yet no single UK governmental institution, body, or regulator currently has the mandate to coordinate action across pesticide regulation, environmental management and clinical AMR policy. A cross-government coordination structure, led by an existing body such as Defra, could establish shared accountability for tackling fAMR—providing a forum to set priorities and manage trade-offs between food security, environmental protection and antifungal efficacy in human medicine.

In practice, responsibilities are distributed across multiple bodies: the Department for Environment, Food and Rural Affairs (Defra) oversees agricultural policy; the Health and Safety Executive (HSE)’s Chemicals Regulation Division (CRD) regulates pesticide approvals; and the AHDB-supported Fungal Resistance Action Group (FRAG-UK) produces guidance on agricultural fAMR. Clinical AMR is led by the Department of Health and Social Care (DHSC), the UK Health Security Agency (UKHSA), Public Health Scotland (PHS), Public Health Wales (PHW) and the Public Health Agency in Northern Ireland (PHA), while environmental regulators and local authorities oversee waste streams. Accountability for fAMR coordination and assessment is, therefore, fragmented. This gap has been raised repeatedly, including in a House of Lords debate in 2023, a written question in 2025, and was reaffirmed at a 2025 House of Lords roundtable on dual-use antifungals, where participants noted the absence of a clear institutional ‘owner’ for fAMR governance^43,44^.

The UK 5-year National Action Plan (NAP) on AMR 2024–2029 articulates a One Health ambition spanning human health, animal health, agriculture and the environment, including fungal AMR. However, the current advisory and delivery mechanisms remain dispersed across multiple specialist committees, and the NAP does not set out the operational steps required to deliver its fAMR commitments^40^. The UK fAMR roadmap similarly identifies governance and coordination gaps as barriers to action. It emphasises that progress on antifungal surveillance depends on optimised diagnostics. Surveillance, in turn, informs resistance-risk assessment and stewardship, which requires joined-up One Health structures rather than isolated technical fixes^9^.

Without a cross-government body tasked explicitly with addressing fAMR, even well-founded technical proposals, such as environmental hotspot monitoring, extended resistance-risk assessment for new fungicides and integrating fungal indicators into national AMR dashboards, are unlikely to gain sustained traction and may remain largely conceptual^9^. While departments and agencies may recognise these risks, accountability is poorly defined, and progress can be restrained by financial limitations in key sectors^9,40^.

Regulatory fragmentation in the post-Brexit landscape may have further complicated the UK’s response to dual-use antifungals. In the EU, an EFSA-led, multi-agency assessment proposed a tiered framework to assess the risk that azole PPPs select resistance in non-target *Aspergillus* spp., explicitly recommending the consideration of environmental hotspots and their role in resistant *A. fumigatus* selection into approval of azoles^11,39^. In the UK, while resistance-risk in target plant pathogens is currently evaluated during pesticide approval^45^, the risk that the use of a specific PPP promotes the emergence and amplification of cross-resistance in human fungal pathogens is not. Therefore, there is a lack of a standardised protocol to generate the data needed for routine evaluation of when and where selection pressures on opportunistic human pathogens, such as *A. fumigatus*, could occur, either for currently approved products or for new PPPs submitted for approval.

The cross-selection risk for new compounds with dual-use modes of action is now well documented. The urgent need for novel modes of action to tackle both plant and human fungal pathogens is demonstrated by the development of dihydroorotate dehydrogenase (DHODH) inhibitors, which represent a new compound class for both crop protection and treatment of human fungal disease. Ipflufenoquin is the first DHODH to be approved for use in agriculture (currently approved in the USA, Australia, Japan, New Zealand, Canada, and under review in the EU), and it shares its mechanism of action with the clinical antifungal olorifim, currently in stage III clinical trials^46^. Worryingly, experimental evidence has shown the potential for ipflufenoquin use to select for olorofim-resistant *A. fumigatus*^47^. In addition, ipflufenoquin has extreme environmental stability, with Dissipation Time 50% (DT50) in laboratory studies ranging from 428 to 1903 days, and in soils up to 767 days^48^, with some reports reporting a half-life of over 7 years^48,49^. Together, the ability of ipflufenoquin to select for resistance and its persistence in the environment pose a significant concern that its use will undermine the clinical efficacy of olorofim, potentially reducing future treatment options for invasive aspergillosis prior to availability. Mirroring this, the prodrug fosmanogepix, a first-in-class antifungal with activity against Candida and Aspergillus species, and difficult-to-treat moulds, which is currently in stage 3 clinical trials^50^, shares a mode of action with aminopyrifen, a novel fungicide for plant pathogenic fungi^51^. Both compounds target fungal glycosylphosphatidylinositol (GPI) biosynthesis, inhibiting the Gwt1 enzyme^52–54^, leading to concern that the dual use of these treatments could lead to cross-resistance^46,55^, however, there is currently no empirical data on this risk.

While we have highlighted the potential risk associated with a single novel DHODH inhibitor, a future dual-use compound with a favourable environmental profile could still pose an fAMR risk if its mode of action overlaps with that of clinical antifungals. Additionally, individual active substances within a class can exert different selective pressures^56^, reinforcing the need to address the resistance selection risk posed by fungicidal products on a case-by-case basis. Although no DHODH-inhibitor fungicides for crop protection have been approved in the UK so far^57^, the UK remains exposed to resistance that may emerge following the agricultural use of DHODH-inhibitors elsewhere. We argue that this threat is exacerbated by the current absence of an explicit requirement in UK pesticide regulatory practice to evaluate or mitigate such cross-resistance risks when approving or re-authorising fungicides.

Without the required institutional reform, the UK is likely to fall behind in managing fAMR, even as the scientific and policy case for action strengthens. The fAMR roadmap proposed by Fisher et al.^9^ organises its recommendations around four priority pillars: risk assessment, surveillance, hotspot management and social and economic drivers. It argues that progress on these pillars will depend on cross-sector governance, mandated surveillance schemes and sustained funding. The Chief Medical Officer’s 2025 report reinforces this message from within the government, calling for research and surveillance monitoring at a time when mycology laboratory and workforce capacity remain constrained, and warning that agricultural deployment of novel fungicides may drive the spread of resistant strains capable of causing serious human disease^40^. On an international level, the WHO FPPL and the WHO Global AMR Research Agenda have elevated fungal pathogens and antifungal resistance to priority status, highlighting major gaps in burden estimates and calling for strengthened surveillance, diagnostics, governance, and sustained research and development investment^3,8,10^. Northern European experts from Denmark, Belgium, Ireland and the UK reported at the NAPP Pharmaceuticals Limited (Mundipharma UK) workshop that WHO FPPL has catalysed discussion about laboratory capacity and surveillance, but also emphasised that there is only patchy surveillance and limited antifungal-resistance monitoring, with significant investment still required to turn priorities into implementable action^3^. Taken together, these national and international assessments make it clear that fungal AMR is structurally under-governed and that recognition without institutional reform will not close the governance gap. Incremental adjustments, such as expedient guidance or short-term pilots, will not be enough. To protect the effectiveness of current and future antifungals, whilst accounting for crop protection’s dependence on current and novel fungicides, including new modes of action, the UK will need a dedicated, cross-government fAMR mechanism with authority to align pesticide regulation, environmental monitoring and clinical AMR policy. It is this institutional change that the recommendations in Section 4 aim to target.

## Recommendations

Addressing fAMR requires structural reforms that match the cross-sectoral pathways through which resistance emerges. The sections above show that environmental selection, clinical outcomes and pesticide regulatory decisions currently operate in isolation. To close this governance gap, we propose three recommendations.

## Establish a cross-government One Health body for fAMR

A dedicated mechanism with a statutory mandate should coordinate consideration of fAMR by Defra, HSE CRD, DHSC, UKHSA, PHS, PHW and PHA and environmental regulators. Such a body would provide a single locus of responsibility for assessing dual-use antifungal risks, brokering trade-offs between agricultural and health priorities, and translating scientific evidence into regulatory decisions. It should also be responsible for setting fAMR criteria for pesticide approvals where a scientifically verified risk of resistance selection in human fungal pathogens from agricultural use exists, and for setting surveillance standards focused on these hotspots. This approach would build on multi-agency frameworks proposed or emerging in the EU^11^ and the US^58^ and would provide a foundation for more coherent UK action.

## Implement a national environmental–clinical surveillance system

The UK should move from research-led sampling to a mandated surveillance scheme that routinely samples known environmental hotspots, as well as baseline fAMR bioaerosols, integrates genomic data from environmental and clinical isolates, and incorporates fungal indicators into AMR dashboards. Existing infrastructures, including PATH-SAFE, national reference laboratories and genomic platforms, provide a viable foundation. Systematic surveillance would supply the empirical baseline required to guide pesticide approvals and pesticide use, waste-management approaches and standards, and clinical preparedness.

## Introduce resistance-risk assessment into fungicide regulation

**Fig. 1:**
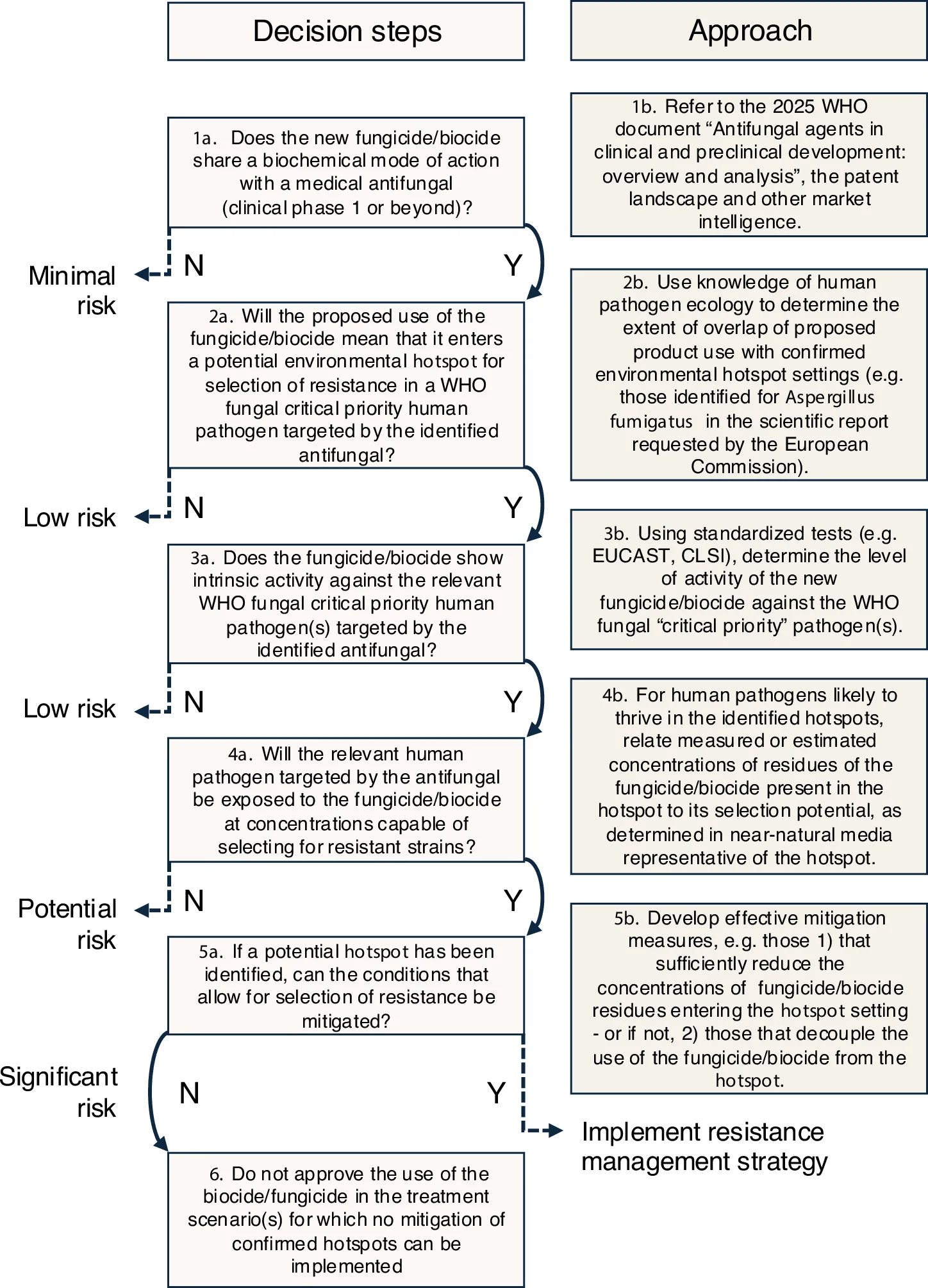
Proposed cross-resistance risk assessment framework for the approval and re-approval of plant protection products (PPPs). While the resistance-risk in target plant pathogens is currently evaluated during pesticide approval in the UK, the risk of cross-resistance in human fungal pathogens is not. This proposed decision cascade provides the basis for extending the current guidelines to explicitly assess human fungal pathogen risk. Major decision steps (left) to consider when assessing the risk of cross-resistance development categorise risk from ‘Minimal risk’ to ‘Significant risk’, while Approaches (right) suggest methodologies for evaluation when considering decision steps. The final decision determines whether a resistance management strategy is required by an applicant or, where mitigation of confirmed hotspots is not possible, a recommendation not to approve the proposed use of the PPP. ‘Y’ and ‘N’ denote ‘Yes’ and ‘No’ outcomes, respectively. In cases where there is substantial uncertainty, assessments should lean towards assuming a higher risk. Environmental ‘hotspots’ are defined as environments that promote the selection, amplification and spread of resistance. While the presented decision cascade is pathogen-agnostic, it should be applied to and validated first for *A. fumigatus*, the pathogen for which we have the most data, before applying it to other pathogens. While the decision cascade currently focuses on the WHO FPPL, allowing for a clear internationally-recognised boundary for assessment, the decision cascade also highlights the need to strengthen our understanding of the ecology of the other clinically relevant fungal pathogens in the environment, as their relative importance, distribution and disease burden will vary geographically.

Extend resistance-risk assessment for the approval and re-approval of PPPs to include explicit evaluation of the potential risk of selecting resistance in opportunistic human pathogens, such as *A. fumigatus*, within environmental hotspots where fungicide exposure is considered a contributing factor to the emergence and spread of resistance (Fig. 1). This must cover the cross-resistance risks for shared modes of action intended for use in both agriculture and medicine. Embedding consideration of antifungal resistance into UK pesticide regulation would align regulatory practice with contemporary scientific understanding and international approaches. A clear, legislated framework would help to reduce uncertainty for developers of new fungicides, help safeguard investment in current and future medical antifungals and support innovation pipelines that deliver novel modes of action.

Together, these recommendations move beyond fragmented, ad hoc responses towards an integrated One Health system capable of detecting, assessing and mitigating fAMR. Implementing them could help to protect the effectiveness of current and future antifungals while positioning the UK to play a leading role internationally on fungal AMR governance.

## Conclusion

fAMR exposes a structural weakness in current UK AMR governance, as existing systems do not integrate environmental, agricultural, veterinary and clinical drivers. Dual-use antifungals create cross-resistance pathways that are not yet adequately addressed by sector-specific policies or technical fixes. The UK is well-positioned to build upon its success in leading bacterial AMR solutions by adopting an integrated One Health approach to fAMR that aligns pesticide regulation, environmental monitoring and clinical AMR policy under a single governance structure, thus exploiting its wealth of expertise in fAMR. Implementing such reforms would contribute towards protecting existing antifungals and safeguarding future antimicrobial innovations whose value depends on maintaining treatment efficacy. Pathogenic fungi, and the spores that they produce, are not bound by political or geographical borders, and while we present UK-centric policy recommendations, the implications of fAMR have a global reach. The dual-use of antifungals, fragmentation across agricultural, environmental and clinical policy domains, and gaps in surveillance and risk assessment are not unique to the UK and are likely to occur in many other settings across the globe. However, the institutional arrangements, regulatory frameworks and resource constraints shaping these challenges will vary between countries. The recommendations outlined here therefore provide a generalisable framework that can be adapted to different national and regional contexts, rather than a prescriptive model tied to a single governance system. With international momentum growing and the scientific evidence becoming increasingly clear, the opportunity is within reach. Treating fAMR as a policy problem in its own right is both achievable and urgent. What remains is the political will to act.

## Data Availability

No datasets were generated or analysed during the current study.
